# Affects as Mediators of the Negative Effects of Discrimination on Psychological Well-Being in the Migrant Population

**DOI:** 10.3389/fpsyg.2020.602537

**Published:** 2020-12-11

**Authors:** Alfonso Urzúa, Diego Henríquez, Alejandra Caqueo-Urízar

**Affiliations:** ^1^Escuela de Psicología, Universidad Católica del Norte, Antofagasta, Chile; ^2^Instituto de Alta Investigación, Universidad de Tarapacá, Arica, Chile

**Keywords:** migrant, well-being, positive affect, negative affect, discrimination, racism

## Abstract

There is abundant empirical evidence on the negative effects of discrimination on psychological well-being. However, little research has focused on exploring the factors that can mitigate this effect. Within this framework, the present study examined the mediating role of positive and negative affects in the relationship between ethnic and racial discrimination and psychological well-being in the migrant population. About 919 Colombians, first-generation migrants, residing in Chile (Arica, Antofagasta, and Santiago) were evaluated, of which 50.5% were women, and the participants’ average age was 35 years (range: 18–65 years). Krieger’s discrimination questionnaires, Watson’s Positive and Negative Affect Schedule (PANAS), and Ryff’s Psychological Well-Being Scale were applied. The measurement models of each variable were estimated, and then the structural equation models were used. The results of the hypothesized multiple mediation model showed that the main mediator in the relationship between ethnic-racial discrimination and psychological well-being was positive affects over negative ones.

## Introduction

Global processes on migration had implied that by mid-2019, about 272 million people would be living outside their countries of birth (Organización de Naciones Unidas, 2019). In South America, by 2019, the number of immigrants had reached almost 10 million, of which nearly a million were in Chile (Organización Internacional para las migraciones, 2020).

Even when migrants move to other countries seeking better living conditions and well-being, they often face different demanding and negative situations, such as living in overcrowded conditions, being victims of sexual or labor exploitation, or other types of violence, which negatively affects their quality of life, mental health, and well-being (Bobowik et al., 2015; Urzúa et al., 2015, 2016, 2017a,b; Bas-Sarmiento et al., 2017; Foo et al., 2018; Gatt et al., 2020).

Especially regarding well-being in the migrant population, evidence suggests that it can be affected by various variables at both individual and contextual levels, such as sex, educational level, age, length of residence, administrative situation, work situation, social support, acculturation strategies, language, positive social interaction, environment, and mental health (Liu et al., 2017; Urzúa et al., 2019a, 2020a; Rodríguez et al., 2020).

Besides these factors, a greater or smaller level of well-being may be conditioned by the individual’s level of adaptation in the host country, a process that is influenced by other factors such as ethnicity, language, religion, or an appearance different from that of the inhabitants of the host country (Martine et al., 2000). Low tolerance of these differences may produce phenomena such as discrimination and segregation by the host country (Tijoux et al., 2011).

Discrimination, a major negative social situation faced by migrants, is conceptualized as a different treatment toward a group with common characteristics or toward a person who belongs to such a group (Krieger, 2001). Discrimination can be exercised in several ways, with ethnic and racial discrimination being the most common among the migrant population. Racial discrimination refers to any differential treatment based on race or skin color. Ethnic discrimination involves situations of inequality and exclusion resulting from belonging to a specific ethnic group, a group that is formed by individuals who are perceived to have a common heritage with a common language, culture, and ancestry (Booth et al., 2011), and who are a minority in the host location.

Not only does discrimination have multiple negative effects on the population that suffers it, ranging from inequalities in access to socioeconomic goods and services and labor sources to access to health and education benefits, but also abundant evidence has revealed its negative effects on individuals’ physical and mental health and well-being (Harrell et al., 2003; Paradies, 2006; Gee et al., 2009; Pascoe and Smart, 2009; Williams and Mohammed, 2009; Bastos et al., 2014; Lewis et al., 2015; Cuevas et al., 2016; Lahoz and Forns, 2016; Williams et al., 2019; Urzúa et al., 2019b, 2020a), especially stigmatized groups such as the migrant population (Finch et al., 2000; Bourguignon et al., 2006; Greene et al., 2006; Borrell et al., 2010; Zeiders et al., 2013; Schunck et al., 2015).

An inverse relationship between discrimination and well-being levels has been reported in studies of both the general population (Schmitt et al., 2014; Castaneda et al., 2015) and migrant population (Jasinskaja-Lahti et al., 2006a,b; Mesch et al., 2008; Sevillano et al., 2013; Giuliani et al., 2018; Stevens and Thijs, 2018; Kader et al., 2020). However, studies on the factors that may moderate or mediate this relationship are still scarce. Factors such as personality traits (Xu and Chopik, 2020), self-esteem (Urzúa et al., 2018), identity (Jasperse et al., 2011; Liu and Zhao, 2016; Ferrari et al., 2017), sense of control (Jang et al., 2008), ethnic affirmation (Atari and Han, 2018), employability (Mera-Lemp et al., 2019), and group membership (Choi et al., 2020) could mediate the relationship between perceived discrimination and psychological well-being, or moderate it, in the case of group effectiveness (Bagci and Canpolat, 2020) or group membership (Shinwoo et al., 2020).

Given its close relationship to both discrimination and well-being, one factor that could play a mediating role is affect or emotional experience (Greenglass and Fiksenbaum, 2009; Pierce et al., 2018). Emotional experience has been divided into two dimensions: one positive and the other negative (Watson and Tellegen, 1985). Precedents suggest that affects can have a mediating role in the relationship between well-being and other factors, such as optimism (Vera-Villarroel et al., 2016). Although from a hedonic perspective, affects together with life satisfaction constitute the primary components of subjective well-being (Diener et al., 1999), there is evidence that the components of this structure behave independently and are moderately related (Busseri, 2018), which would also be expected for a measure of well-being, but from an eudaimonic perspective, as is psychological well-being (Ryff, 1989, 2014).

This research is framed in the context of south-south immigration, that is, South Americans migrating to South American countries, and specifically Colombians to Chile. Colombian migration mainly derives from the Pacific coast, i.e., people of African descent. This migration has resulted in situations of discrimination, either by the country of origin, linked to drug trafficking, drugs, and sex trade in the case of women, or by the color of the skin (Pavez, 2016; Tijoux, 2016; Gissi et al., 2019). Studies conducted in Chile show how discrimination has negatively affected both the mental health and the well-being of this population (Urzúa et al., 2018, 2019b; Mera-Lemp et al., 2020a), in addition to other factors that also affect well-being (Silva et al., 2016; Urzúa et al., 2019a, 2020b; Mera-Lemp et al., 2020b). A qualitative perspective about the effects of migration and racism on the Colombian population in Chile can be reviewed in Gii-Barbieri and Ghio-Suárez (2017) and Gii et al. (2019).

**Figure 1:**
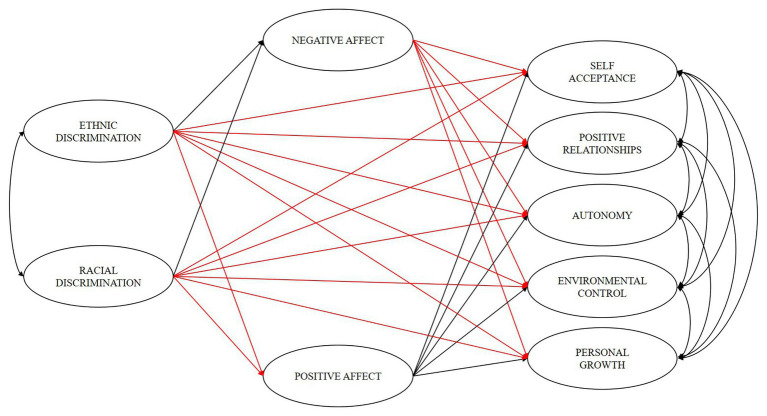
Hypothetical model. Positive affects are shown in black and negative affects in red.

In this framework, this study examined the mediating role of positive and negative affects in the relationship between ethnic and racial discrimination and psychological well-being in Colombian migrants living in Chile. Based on the literature review, we hypothesized that the relationship between discrimination (racial and ethnic) and psychological well-being (self-acceptance, positive relationships, autonomy, environmental mastery, and personal growth) would be mediated by both positive and negative affects (Figure 1).

## Materials and Methods

### Participants

We surveyed a total of 919 migrants of Colombian nationality, who were living in three cities with the highest number of registered migrants in Chile: 476 (51.8%) in Antofagasta, 219 (23.8%) in Arica, and 224 (24.4%) in Santiago, at the time of the survey. It should be noted that the Metropolitan and the Antofagasta are the two regions with the highest number of visas issued by 2018 (Departamento de Extranjería y Migración, 2020). Regarding gender, 455 (49.5%) identified themselves as men, and 464 (50.5%) were women. Participants’ age ranged from 18 to 65 years (ME = 35.27; SD = 9.91). The characteristics of the participants can be observed in Table 1.

**Table 1:** Sociodemographic characteristics of participants.

Variables	*n* (%)
Sex	
Male	455 (49.5)
Female	464 (50.5)
City	
Arica	219 (23.8)
Antofagasta	476 (51.8)
Santiago	224 (24.4)
Years of arrival in Chile^a^	
>10 years	40 (4.4)
1–10 years	854 (92.9)
Does not respond	25 (2.7)
Education^a^	
Incomplete primary education	102 (11.1)
Primary education	233 (25.4)
Secondary education	309 (33.6)
Incomplete technical education	82 (8.9)
Technical level	116 (12.6)
Incomplete University education	37 (4.0)
University education	20 (2.2)
Postgraduate	6 (0.7)
Does not respond	14 (1.5)
Legal situation^a^	
With residence Visa	681 (74.1)
Without residence Visa	117 (12.7)
Nationalized	61 (6.6)
Does not respond	60 (6.5)
Employment^a^	
Employee	656 (71.4)
Retired	4 (0.4)
Unemployed	122 (13.3)
Housewife	59 (6.4)
Student	33 (3.6)
Does not respond	45 (4.9)
Monthly income^a^	
<125 US$	112 (12.2)
126–375 US$	331 (36.0)
376–750 US$	355 (38.6)
751–1,250 US$	83 (9.0)
1,251–1,875 US$	8 (0.9)
>1,876 US$	7 (0.8)
Does not respond	23 (2.5)
Self-reported phenotype	
White	197 (21.4)
Indigenous	38 (4.1)
Mestizo	219 (23.8)
Afro-descendant	216 (23.5)
Mulatto	161 (17.5)
Others	14 (1.5)
Does not respond	74 (8.1)

a Variables with lost data.

### Instruments

#### Discrimination

We used Krieger et al. (2005) Discrimination Experience Scale to assess discrimination. This scale measures the participants’ perception of the various situations they have experienced related to discrimination in different contexts. To measure racial and ethnic discrimination separately, we asked the participants about their experiences where a treatment was perceived as discriminatory, whether due to skin color or nationality. Each scale is composed of nine items that ask if the person has felt discriminated, for example, when requiring attention in a restaurant or service, with answers ranging from never (0 points) to four or more times (3 points). In this application, an alpha of 0.88 was obtained for the ethnic discrimination scale and 0.90 for the racial discrimination scale.

#### Affect

It was evaluated using the PANAS, a self-report scale comprising two dimensions designed to measure positive and negative affects (Watson et al., 1988). The scale contains 20 items describing a series of feelings and emotions, and the participants indicate the extent to which they usually or regularly feel these affects with response options ranging between 1 (never) and 5 (very much). The present study used the Chilean version of Vera-Villarroel et al. (2019) and obtained Cronbach’s alphas of 0.87 for both positive and negative affects.

#### Psychological Well-Being

The Spanish adaptation of the Psychological Well-Being Scale of Ryff (1989) was used to measure psychological well-being (Díaz et al., 2006). This version includes 29 items under six dimensions: self-acceptance, positive relationships, autonomy, environmental mastery, purpose in life, and personal growth. Responses are rated on a six-point Likert scale ranging from 1 = totally disagree to 6 = totally agree. There is evidence of its reliability and validity based on the internal structure of the measurement instrument (Chitgián-Urzúa et al., 2013; Vera-Villarroel et al., 2013). In this study (considering the results of the fit of the measurement model prior to the realization of the SEM), a reduced version of the scale was used. This short version contained 17 items under six dimensions proposed by Ryff. However, the “purpose” dimension was not used because it presented anomalous correlations (*r* > 1.0). In the present study, the scale presented Cronbach’s alphas of 0.79 for self-acceptance, 0.70 for positive relationships, 0.77 for autonomy, 0.62 for environmental mastery, and 0.81 for personal growth.

### Procedures

This study is part of a larger project studying the effects of discrimination on health and well-being, which has been reviewed and approved by the Scientific Ethics Committee of the Catholic University of the North. The initial participants were interviewed in person, mainly in public institutions such as the Catholic Migration Institute of Chile (INCAMI), Global Citizen-Jesuit Migration Services, Immigration Department, the Colombian Consulate, health centers, among others, after signing an informed consent. The data were coded and analyzed using SP-21 software.

#### Statistical Analysis

First, the measurement models of each scale were estimated using confirmatory factor analysis. Second, a structural equation model (SEM) was used to test whether ethnic discrimination (ED) and racial discrimination (RD) exerted an inverse effect on migrants’ psychological well-being. Subsequently, the hypothesized multiple mediation model was evaluated, where the mediating effect of positive and negative affects was estimated on the relationship between ethnic and racial discrimination (as a criterion variable) and self-acceptance, positive relations, autonomy, environmental mastery, and personal growth (as response variables). The indirect effects of the mediation model were aeed following the recommendations of Stride et al. (2017).

Structural equation models were performed using Mplus 8.2 software (Muthén and Muthén, 2010), using the weighted least squares (WLSMV) robust estimation method, which is robust for non-normal ordinal variables (Beauducel and Herzberg, 2006). Goodness-of-fit of all models was estimated using Chi-square values (χ^2^), the approximation mean square error (RMSEA), the comparative fit index (CFI), and the Tucker Lewis index (TLI). According to the recommended literature standards (e.g., Schreiber, 2017), the RMSEA ≤ 0.08, CFI ≥ 0.95, and TLI ≥ 0.95 values are considered adequate and indicative of a good fit. Age, sex, city of residence, and self-reported phenotype were controlled for in all analyses. No significant differences were found in the levels of perceived well-being given the voluntary or forced nature of migration, so the analyses did not consider this variable as a control.

## Results

### Measurement Models

Table 1 shows the goodness-of-fit indices of the estimated measurement models. Both ED and RD presented indicators outside the recommended standards (i.e., RMSEA > 0.08). The items that could be causing the poor fit were examined, and it was detected that the items “On being hired or getting a job” and “On the job” could be sharing more variance than was directly explained by the common factor (Lloret-Segura et al., 2014) because both items indicated the work setting. For this reason, we evaluated both the measurement models by extracting the reagent “Upon being hired or obtaining a job,” leaving the reagent “At work” only. With this modification in both scales, the adjustment indicators were close to those recommended by the literature (ED: RMSEA = 0.08; CFI = 0.98; TLI = 0.97; RD: RMSEA = 0.09; CFI = 0.98; TLI = 0.97; Table 2).

**Table 2:** Indicators of global fit of the measurement models and the multiple mediation model.

Models	Parameters	χ^2^	DF	*p*	CFI	TLI	RMSEA	RMSEA IC 90%	
								Low	Superior
ED	36	325.459	27	0.00	0.957	0.943	0.111	0.101	0.122
ED^*^	32	137.687	20	0.00	0.981	0.973	0.081	0.069	0.094
RD	38	234.512	27	0.00	0.973	0.965	0.096	0.085	0.107
RD^*^	34	157.286	20	0.00	0.981	0.973	0.090	0.078	0.104
PANAS	96	615.577	151	0.00	0.965	0.961	0.059	0.054	0.064
PA	50	390.376	35	0.00	0.951	0.938	0.107	0.098	0.117
NA	45	238.282	27	0.00	0.972	0.962	0.094	0.083	0.105
RYFF	102	2,860.721	362	0.00	0.727	0.694	0.085	0.082	0.088
RYFF^*^	130	731.668	109	0.00	0.963	0.954	0.080	0.074	0.085
SEM	227	1,961.586	586	0.00	0.939	0.932	0.055	0.052	0.058
Mediation	334	3,027.991	1,312	0.00	0.941	0.936	0.041	0.039	0.043

ED, ethnic discrimination; RD, racial discrimination; SEM, structural equation model; PA, positive affect; NA, negative affect.

* Clean models.

### Structural Equation Model

Based on the adjusted measurement models, a structural equation model was used to examine the effects of ED and RD on the components of psychological well-being (self-acceptance, positive relationships, autonomy, environmental mastery, and personal growth). In regard to the control variables, we could only observe significant effects of self-reported phenotype on Personal Growth (*b* = 0.10), and the city of residence on Self-acceptance (*b* = 0.09), Positive Relationships (*b* = 0.19), and autonomy (*b* = −0.12). Age and sex did not present significant effects on the dimensions of psychological well-being.

**Figure 2:**
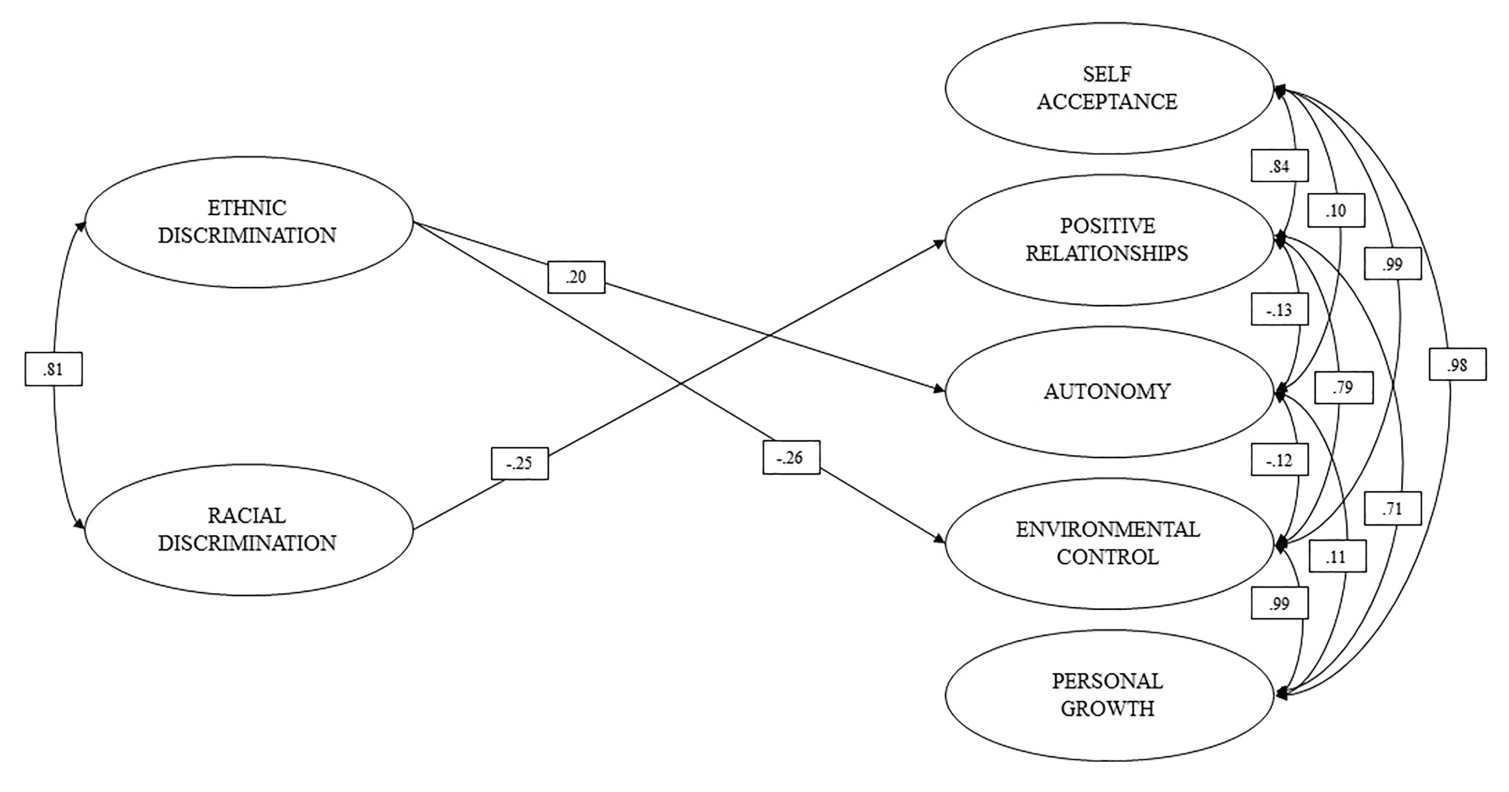
Relationship between discrimination and well-being.

As shown in Figure 2, ED had a slight positive effect (*b* > 0.10; Cohen, 1988) on autonomy, a small negative effect on environmental mastery, and no significant effect on self-acceptance, positive relationships, and personal growth.

On the other hand, RD exerted a slight negative effect on positive relationships (*b* = −0.251; *p* < 0.00) and a moderate negative effect (*b* > 0.30; Cohen, 1988) on personal growth (*b* = −0.321; *p* < 0.00). RD did not have significant effects on self-acceptance, autonomy, and environmental mastery. The structural model presented goodness-of-fit close to the criteria accepted in the literature (RMSEA = 0.055; CFI = 0.939; TLI = 0.932).

Once the relationship between ethnic-racial discrimination and psychological well-being was examined (Table 3), the model of multiple mediations was evaluated. In this model, the positive and negative effects on migrants were included as parallel mediators of the inverse effect that ED and RD would have on self-acceptance, positive relations, autonomy, mastery of the environment, and personal growth. In regard to the control variables, we could only observe significant effects of sex on positive affects (*b* = −0.09), self-reported phenotype on Personal Growth (*b* = 0.09), and city of residence on positive affects (*b* = 0.21), Positive Relationships (*b* = 0.13) and autonomy (*b* = −0.19). Age did not present significant effects on positive/negative affects, or on dimensions of psychological well-being.

**Table 3:** Scores of the variables included in the model.

Variables	n	ME	SD	PR	AU	EM	PG	ED	RD	PA	NA
PWB											
SA	896	5.33	1.21	0.63^**^	0.09^**^	0.69^**^	0.77^**^	−0.17^**^	−0.14^**^	0.36^**^	−0.18^**^
PR	887	4.86	1.33		−0.70^*^	0.52^**^	0.53^**^	−0.19^**^	−0.19^**^	0.26^**^	−0.12^**^
AU	902	4.20	1.50			−0.08^*^	0.08^*^	0.08^*^	0.02	0.23^**^	−0.09^*^
EM	899	5.13	1.29				0.69^**^	−0.17^**^	−0.12^**^	0.25^**^	−0.13^**^
PG	908	5.46	1.36					−0.13^**^	−0.13^**^	0.32^**^	−0.19^**^
Discrimination											
ED	856	0.53	0.65						0.70^**^	−0.08^*^	0.25^**^
RD	810	0.40	0.61							−0.13^**^	0.23^**^
Affects											
PA	903	3.42	0.86								−0.10^**^
NA	908	1.93	0.74								

PWB, psychological well-being; ED, ethnic discrimination; RD, racial discrimination; PA, positive affect; NA, negative affect; SA, self-acceptance; PR, positive relationships; AU, autonomy; EM, environmental mastery; PG, personal growth.

** *p* < 0.01;

* *p* < 0.05.

**Figure 3:**
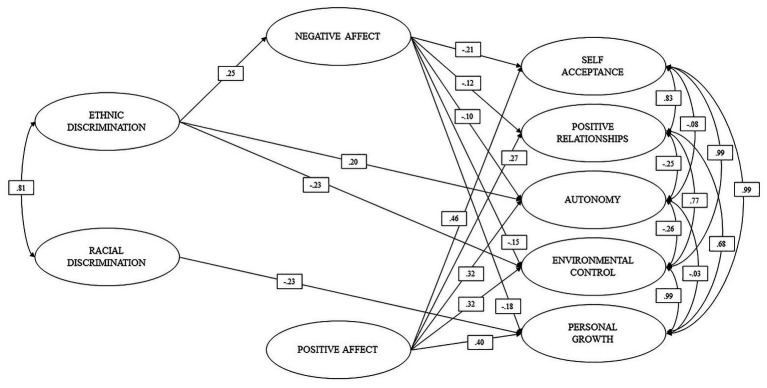
Model of multiple mediations.

Figure 3 shows the significant direct effects of the mediation model. As can be seen, ethnic discrimination presented positive direct effects of small magnitude on negative affects and autonomy, and a negative effect on mastery of the environment. No significant effects were observed on the other variables. Regarding ethnic discrimination, only small and significant negative direct effects can be observed on personal growth, nor were there significant effects observed on the other variables. Furthermore, in Figure 3, it can be seen that negative affects exert small negative direct effects on self-acceptance, positive relationships, autonomy, mastery of the environment, and personal growth, while positive affects present positive direct effects of moderate magnitude on self-acceptance, positive relationships, autonomy, control of the environment, and personal growth.

As shown in Table 4, negative affect only exerted indirect mediation effects (Zhao et al., 2010) on the relationship between ED and self-acceptance and personal growth, and complementary effects (Zhao et al., 2010) on the relationship between ED and environmental mastery. Positive affect did not have significant mediation effects. In addition, total indirect effects can be observed on the relationship between RD and self-acceptance, positive relationships, environmental mastery, and personal growth.

**Table 4:** Standardized indirect and total effects of the mediation model.

Effects	Direct	Indirect NA	Indirect PA	Indirect total	Total
ED → NA/PA → SA	−0.051	−0.051^*^	0.024	−0.027	−0.079
ED → NA/PA → PR	0.012	−0.030	0.014	−0.016	−0.003
ED → NA/PA → AU	0.203^*^	−0.026	0.017	−0.009	0.194^*^
ED → NA/PA → EM	−0.233^*^	−0.036^*^	0.017	−0.020	−0.253^*^
ED → NA/PA → PG	0.134	−0.044^*^	0.021	−0.024	−0.111
RD → NA/PA → SA	−0.058	−0.020	−0.079	−0.099^*^	−0.157
RD → NA/PA → PR	−0.192	−0.012	−0.048	−0.059^*^	−0.251^*^
RD → NA/PA → AU	−0.046	−0.010	−0.056	−0.066	−0.112
RD → NA/PA → EM	0.082	−0.014	−0.056	−0.070^*^	0.012
RD → NA/PA → PG	−0.233^*^	−0.017	−0.069	−0.087^*^	−0.320^*^

ED, ethnic discrimination; RD, racial discrimination; PA, positive affect; NA, negative affect; SA, self-acceptance; PR, positive relationships; AU, autonomy; EM, environmental mastery; PG, personal growth.

* *p* < 0.05.

The hypothesized mediation model was adequately adjusted to the data (RMSEA = 0.041; CFI = 0.941; TLI = 0.936); therefore, it was a good representation of the observed relationships.

## Discussion

This study hypothesized that the relationship between discrimination (racial and ethnic) and psychological well-being (self-acceptance, positive relationships, autonomy, environmental mastery, and personal growth) would be mediated by both positive and negative affects, which was partially proven.

First, after controlling for sex, age, city, and self-reported phenotype, the data provide evidence for the fact that discrimination had an effect on well-being. Particularly, ethnic discrimination affected environmental mastery, and racial discrimination affected positive relationships. Concurrently, a slight positive effect of ethnic discrimination on autonomy was found.

The inverse relationship of xenophobia with the environmental mastery is evident, since the migrant, perceiving unequal treatment because one belongs to a specific group (in this case, being Colombian) or according to what one believes or thinks one deserves, diminishes their sense of control over the world and the ability to influence the context around them. Similarly, feeling discriminated against because of the color of one’s skin has a negative effect on the acquisition of stable social relationships and trustworthy friends, especially in a highly racist context, such as Chile, where about one in three people consider themselves whiter than other people in Latin American countries (Instituto Nacional de Derechos Humanos, 2017).

Despite this, feeling discriminated against due to ethnic origin has a direct relationship with the domain of autonomy. This implies that, in some way, discrimination has generated a higher level of autonomy, which allows migrants to better resist social pressure and sustain their individuality in different social contexts, where both self-esteem and ethnic identity play an important role (Urzúa et al., 2018).

Second, regarding the incorporation of affects, whether positive or negative, in the relationship between discrimination and the domains of psychological well-being, there is evidence of a positive relationship between positive affects and well-being and an inverse relationship between negative affects and well-being, in a similar way to the relationship between affections and hedonic well-being. It should be noted that when the affects are introduced into the model, the relationships between ED and autonomy, and environmental control are maintained; however, the relationship between RD and positive relationships disappears, and the relationship with personal growth appears. The latter seems to provide evidence on the effect that the presence of both affects may have in the relationship of racial discrimination on personal growth.

However, we also found that affects effectively exercised a mediating role. Negative affect only exerted indirect mediation effects on the relationship between ED and self-acceptance and personal growth, and complementary effects on the relationship between ED and environmental mastery.

Complementary mediation occurs when the mediated effect (a × b) and the direct effect (c) exist, and point in the same direction (Zhao et al., 2010). This means that ethnic discrimination has negative effects on the environmental mastery, and in parallel, negative affects also have negative effects on this domain. This means that migrants who perceive greater discrimination due to their country of origin are affected in their abilities to be able to deploy and modify the context and environment for their benefit.

We have also found that the presence of both affects had a mediating effect on the relationship between RD and self-acceptance, positive relationships, environmental mastery, and personal growth. This is relevant since, although specific indirect effects can be observed in the multiple mediation model, it is very difficult for these effects to work separately in real life. Therefore, it is necessary to understand the phenomenon in a more complete and holistic way considering the inclusion of both negative and positive affects simultaneously. The results indicate that positive/negative affects as a whole play a fundamental role in explaining the low levels of psychological well-being caused by racial discrimination. These results open the need to deepen a possible line of study that allows us to continue enhancing our understanding of the effect of affects on the discrimination-well-being relationship, which could be to consider the variables of positive or negative affect not as independent variables but as a single variable elaborated based on the combination of both. Pierce et al. (2018) research could be considered as a precedent. They analyzed the mediating effect of affect on ethnic and racial minorities in African American students in the United States, showing that affect mediated well-being, but specifically, the combination of positive and negative affect, where high positive affect combined with low negative affect was associated with improved well-being. Similarly, it would be interesting to explore how this relationship might be affected by a possible moderating variable, such as sex, given that women’s stronger positive emotions have been reported to balance their greater negative affects (Fujita et al., 1991).

Therefore, the discussion focuses on an aspect that deserves our attention. There would be a differentiated effect of the interaction between the types of affects and the types of discrimination, given that the positive affects only presented the capacity to mediate the effect of racial discrimination on some domains of well-being, while the negative affects mediated the effects of ethnic discrimination on well-being only. It was difficult to find literature or previous research that contribute to the discussion about the relationship between the origin of discrimination (by nationality or skin color) and the affects, whether positive or negative. However, undoubtedly, by behaving independently, these would play a different role according to the origin of the discrimination, which opens an interesting line to explore. Xu et al. (2015) have also reported that attentional bias influenced affect and, therefore, the well-being (attention bias to positive information favors positive affects, and the positive affect could reduce the negative cognitive bias induced by negative affect and, therefore, would contribute to better psychological well-being). Racial discrimination would be linked to an attentional bias centered on positive affects, while xenophobia would activate activation bias oriented toward negative affects, thus, opening an interesting line of research.

Taking the above into consideration, and to better understand the phenomenon, by including in the model both positive and negative affects experienced by migrants, it was found that ethnic discrimination (because it comes from a particular country) causes negative affects, and these affects, in turn, cause lower levels of psychological well-being in all its dimensions. In other words, negative affects have a strong influence on the effect that ethnic discrimination has on the psychological distress of migrants. On the other hand, in the case of positive affects, these were not influenced by discrimination or by coming from a particular country, or by having a particular skin color, so this does not influence the relationship to a great extent between ethnic/racial discrimination and dimensions of psychological well-being. However, this does not mean that positive affects have no effects on psychological well-being, quite the contrary. The results show that the positive affects of migrants cause higher levels of psychological well-being for them.

Despite the limitations of this study, which are typical of a cross-sectional study in which the effect of affects over time cannot be evaluated and is performed in only one ethnic group, it is a contribution since much of the evidence found on well-being is based on measuring hedonic well-being (subjective well-being) and not eudemonic well-being (psychological well-being). It also opens new lines of discussion on the differential impact that discrimination due to different causes can have, while reinforcing the evidence of the independent effect of affects, whether negative or positive, on psychological well-being. This merits its inclusion as a variable in the development of intervention programs at the individual level, favoring the psychological well-being of the migrant population.

## Data Availability Statement

The raw data supporting the conclusions of this article will be made available by the authors, without undue reservation.

## Ethics Statement

The studies involving human participants were reviewed and approved by Comité de Ética Científica de la Universidad Católica del Norte. The patients/participants provided their written informed consent to participate in this study.

## Author Contributions

AU and AC made substantial contributions to the conception and design, and acquisition of data. AU and DH performed the analysis and interpretation of the data. All authors participated in drafting the article or revising it critically for important intellectual content, and all authors gave final approval of the version to be submitted.

### Conflict of Interest

The authors declare that the research was conducted in the absence of any commercial or financial relationships that could be construed as a potential conflict of interest.
